# Editorial: Nanoparticles assisted improvement of semen quality in livestock

**DOI:** 10.3389/fvets.2026.1789183

**Published:** 2026-03-17

**Authors:** Rupali Rautela, Rahul Katiyar, Mahak Singh

**Affiliations:** 1 Divison of Cattle Physiology and Reproduction, Central Institute for Research on Cattle, Meerut, India; 2 ICAR Research Complex for North Eastern Hill Region, Umiam, India; 3 ICAR Research Complex for North Eastern Hill Region Nagaland Centre, Jharnapani, India

**Keywords:** antimicrobial resistance, cryopreservation, reactive oxygen species, semen quality, silver nanoparticle

The limited availability of high-quality semen necessitates the efficient utilization of available semen to optimize reproductive outcomes. Minimizing the discard rate of poor-quality semen can significantly improve the utilization of elite sires and, therefore, enable the more widespread dissemination of superior germplasm. Nanoparticle-assisted spermatozoa selection is a cutting-edge technique that shows promise in the field of semen biology. In this context, the use of nanoparticles for spermatozoa selection has emerged as a promising approach to improve semen utilization efficiency in animals. Nanoparticle-based selection techniques enable the targeted isolation of functionally competent spermatozoa based on specific biophysical and biochemical characteristics, such as membrane integrity, surface charge, motility, and DNA integrity. This approach aims to enrich semen doses with high-fertility sperm while reducing the discard rate of poor- or marginal-quality ejaculates. Consequently, nanoparticle-assisted sperm selection has the potential to enhance fertilization efficiency, improve conception rates, and facilitate the wider dissemination of superior germplasm from elite sires in both field and laboratory conditions. Further exploration of this technique is necessary. This research Topic, entitled “*Emerging technologies in semen processing and quality enhancement*” includes three studies contributed by various researchers who explored techniques to maximize quality semen production.

Li et al. reported on the promising effects of glutathione (GSH) supplementation in combination with selenium nanoparticles (SeNPs) on improving the quality of cryopreserved bull semen. Both selenium and GSH possess antioxidant properties, and their combined use may exert a synergistic effect. As a result, semen quality parameters such as motility, viability, mitochondrial activity, plasma membrane integrity, acrosome integrity, and antioxidant activity were significantly improved in cryopreserved bull spermatozoa. In addition, embryonic development potential was enhanced following supplementation with 4 mM GSH and 2 μg/mL SeNPs. However, further studies are needed to confirm the bioactivities of selenium nanomaterials and to evaluate the *in vivo* fertility of semen cryopreserved using this supplementation strategy.

A study on bull semen by Kanwar et al. demonstrated the antioxidant activity of silver nanoparticles (AgNPs) in improving semen quality and reproductive performance by reducing oxidative stress and thereby enhancing sperm function. Of the various doses tested, 20 μg/mL produced the most significant improvements in semen quality, with a marked reduction in morphological abnormalities. These findings suggest that AgNPs may serve as promising semen additives for improving the quality and fertility of cryopreserved bull semen.

The uncontrolled use of antibiotics, leading to antimicrobial resistance, has emerged as a serious concern for both human and animal health. Basioura et al. aimed to examine the effects of insemination without antibiotics on the future reproductive efficiency of sows. In this approach, a single-layer centrifugation protocol using a low-density colloid was employed as an alternative to antibiotic-containing extenders. This method effectively minimized bacterial contamination while achieving fertility outcomes comparable to those obtained with extenders containing antibiotics. The authors concluded that although the technique is time-consuming and incurs additional costs for semen dose production, the extra time and expense are justified in light of the urgent need to identify viable alternatives to antibiotics.

Improving the quality of cryopreserved semen and maximizing the use of genetically valuable sires are major priorities for livestock breeders. Elevated levels of reactive oxygen species (ROS) in semen damage healthy and viable spermatozoa, thereby reducing sperm quality. Nanotechnology has emerged as a transformative frontier in semen biology, offering high- precision tools to enhance sperm quality, select gametes, and preserve them long-term. The antioxidant properties of certain nanoparticles, such as selenium and silver, can reduce oxidative stress, thereby improving post-thaw semen quality and fertility. In addition, the inclusion of antibiotics in semen extenders raises concerns due to the increased risk of antimicrobial resistance. Methods that reduce or remove bacterial contamination in extended semen may enable the use of antibiotic-free extenders. We firmly believe that the application of nanotechnology, along with antibiotic-alternative approaches, will contribute to improved semen quality and fertilizing ability.

